# Reduced Interleukin-4 Receptor α Expression on CD8^+^ T Cells Correlates with Higher Quality Anti-Viral Immunity

**DOI:** 10.1371/journal.pone.0055788

**Published:** 2013-01-31

**Authors:** Danushka K. Wijesundara, David C. Tscharke, Ronald J. Jackson, Charani Ranasinghe

**Affiliations:** 1 The Molecular Mucosal Vaccine Immunology Group, The Department of Immunology, The John Curtin School of Medical Research, The Australian National University, Acton, Canberra, Australia; 2 Viruses and Immunity Laboratory, Division of Biomedical Science and Biochemistry, The Research School of Biology, The Australian National University, Acton, Canberra, Australia; University of Melbourne, Australia

## Abstract

With the hope of understanding how interleukin (IL)-4 and IL-13 modulated quality of anti-viral CD8^+^ T cells, we evaluated the expression of receptors for these cytokines following a range of viral infections (e.g. pox viruses and influenza virus). Results clearly indicated that unlike other IL-4/IL-13 receptor subunits, IL-4 receptor α (IL-4Rα) was significantly down-regulated on anti-viral CD8^+^ T cells in a cognate antigen dependent manner. The infection of gene knockout mice and wild-type (WT) mice with vaccinia virus (VV) or VV expressing IL-4 confirmed that IL-4, IL-13 and signal transducer and activator of transcription 6 (STAT6) were required to increase IL-4Rα expression on CD8^+^ T cells, but not interferon (IFN)-γ. STAT6 dependent elevation of IL-4Rα expression on CD8^+^ T cells was a feature of poor quality anti-viral CD8^+^ T cell immunity as measured by the production of IFN-γ and tumor necrosis factor α (TNF-α) in response to VV antigen stimulation *in vitro*. We propose that down-regulation of IL-4Rα, but not the other IL-4/IL-13 receptor subunits, is a mechanism by which CD8^+^ T cells reduce responsiveness to IL-4 and IL-13. This can improve the quality of anti-viral CD8^+^ T cell immunity. Our findings have important implications in understanding anti-viral CD8^+^ T cell immunity and designing effective vaccines against chronic viral infections.

## Introduction

Protection against intracellular pathogens (e.g. viruses and parasites) and tumors often requires the activation of effector CD8^+^ T cells, which usually mediate cytolysis upon recognition of non-self peptide-major histocompatibility complex class I complexes presented on the surface of malignant cells or virus-infected cells [1]–[3]. However, a collection of CD8^+^ T cell effector functions, not exclusively cytolysis, appears to be important in controlling virus infections. These include the ability to mount high avidity interactions with virus-infected targets, produce multiple anti-viral cytokines (i.e. IFN-γ and TNF-α), induce a high clonal turnover rate, and/or the ability to produce chemoattractants (e.g. macrophage inflammatory protein-1β) to recruit immune cells to virus-infected sites [4]–[6]. These effector functions define the quality of effector CD8^+^ T cell responses against viruses.

IL-4 and IL-13 are closely related cytokines mostly studied for their involvement in allergic diseases (e.g. asthma) and parasitic infections. In our previous studies we have for the first time demonstrated that both IL-4 and IL-13 dampen the avidity of human immunodeficiency virus (HIV)-specific CD8^+^ T cells in a pox-viral prime-boost vaccination model in mice [7]–[8]. Other studies also have shown that exposure of CD8^+^ T cells to IL-4 during primary activation results in the differentiation of effector CD8^+^ T cells with excessively reduced CD8 co-receptor expression, poor cytotoxic capacity, poor IFN-γ production and enhanced IL-4 production *in vitro* and *in vivo* [9]–[11].

IL-4 and IL-13 share many biological functions due to their ability to signal through a unique network of complex receptors. IL-4 can signal through the type I IL-4 receptor (heterodimer of IL-4Rα and common γ (γc) chains) and the type II IL-4 receptor (heterodimer of IL-4Rα and IL-13 receptor α 1 (IL-13Rα1) chains), but IL-13 only signals through the type II IL-4 receptor [12], [13]. IL-4 and IL-13 cytokine responses can be completely abrogated on cells lacking IL-4Rα expression as it is a component of both type I and type II IL-4 receptors [14]–[15]. IL-13 binds with higher affinity to IL-13 receptor α 2 (IL-13Rα2) compared to IL-13Rα1, but binding of IL-13 to IL-13Rα2 does not result in signaling [16]. IL-13Rα2 has been shown to be important in dampening IL-13 signaling and protecting mice against allergen induce dermatitis [17]. Hence, IL-13Rα2 is thought to function as an IL-13 decoy/inhibitor receptor. IL-4 and IL-13 cellular signaling via the type I or type II IL-4 receptors leads to activation of STAT6, which facilitates transcription of IL-4/IL-13 responsive genes [18]–[19].

The use of IL-4Rα ^−/−^, IL-13Rα1 ^−/−^, γc ^−/−^ and IL-13Rα2 ^−/−^ mice have shown that these receptors are indeed important for controlling functions of IL-4 and IL-13 in allergic diseases and parasitic infections [15], [17], [20]. However, these studies provide limited insight as to how these receptors are regulated at a cellular level *in vivo* during the course of a pathogenic infection. Recent study by Tanaka *et al* [21] reported that cell surface IL-4Rα expression is down-regulated on activated CD4^+^ T cells *in vivo* following *L. major* infection. This was found to be due to degradation of IL-4Rα in intracellular compartments of activated CD4^+^ T cells in a T cell receptor and dedicator of cytokinesis 2 dependent manner [21]. In another study, Perona-Wright *et al* [22] have also shown that cell surface IL-4Rα expression was down-regulated on activated CD4^+^ T cells following *H. polygyrus* infection of mice, which was thought to render these cells refractory to further stimulation with IL-4. On the contrary, naïve bystander CD4^+^ T cells in this instance were found to up-regulate IL-4Rα making them more responsive to IL-4 [22]. These studies suggest that IL-4Rα play a critical role in tuning responsiveness of CD4^+^ T cells to IL-4 and/or IL-13 during infection with pathogens.

Despite recent studies showing the importance of regulating IL-4Rα expression on CD4^+^ T cells following parasitic infection *in vivo*, only few studies have addressed how the cytokine receptors for IL-4/IL-13 are regulated on CD8^+^ T cells following virus infection *in vivo*. As our previous studies have shown that IL-4 and IL-13 play an important role in modulating the quality of anti-viral CD8^+^ T cell immunity [7]–[8], in this study we further investigated how IL-4Rα, γc, IL-13Rα1 and IL-13Rα2 were regulated on CD8^+^ T cells and other immune cells predominantly following VV infection of mice. Results indicated that IL-4Rα expression, but not γc, IL-13Rα1 or IL-13Rα2 was differentially regulated on CD8^+^ T cells as a consequence of VV infection of mice. Therefore, the current study focused on the mechanisms involved in the regulation of IL-4Rα on CD8^+^ T cells following virus infection and whether differential regulation of IL-4Rα on CD8^+^ T cells affects the quality of anti-viral CD8^+^ T cell immunity.

## Results

### Regulation of IL-4/IL-13 receptor components during VV infection

**Figure 1:**
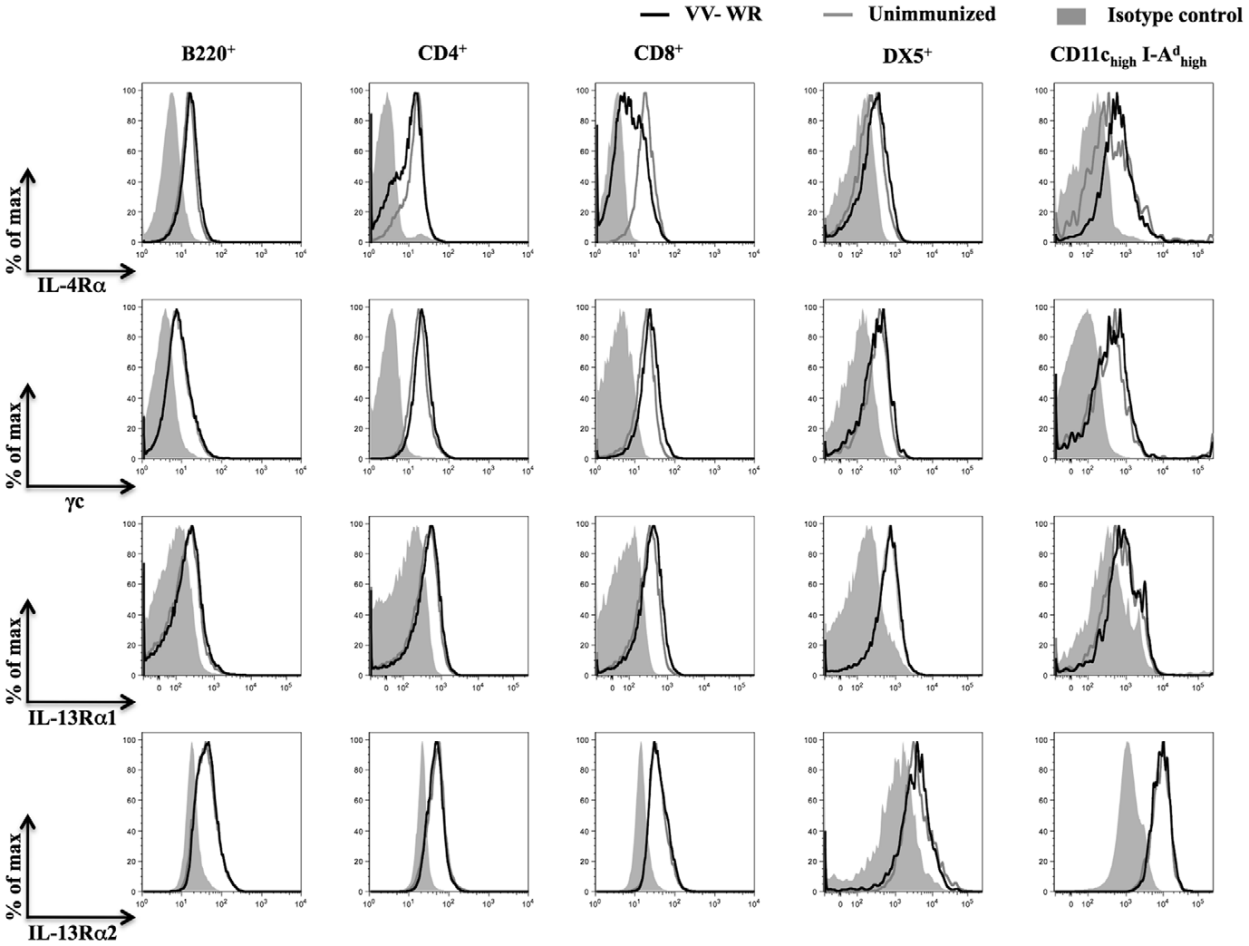
Distribution of IL-4Rα, γc, IL-13Rα1 and IL-13Rα2 on immune cells following VV infection. BALB/c WT mice (n = 12) infected for 7 days with VV-WR or unimmunized were sacrificed and splenocytes harvested for analysis using flow cytometry. The histogram plots show cell surface expression of IL-4Rα, γc, IL-13Rα1 and intracellular expression of IL-13Rα2 on gated B220^+^, CD4^+^, CD8^+^, DX5^+^, and CD11c_high_ I-A^d^_high_ splenocytes from a representative VV-WR infected mouse (black lines) and an unimmunized mouse (grey lines). The plots are representative of at least 12 mice tested in at least three independent experiments.

To examine whether VV infection induced differential regulation of IL-4/IL-13 receptor subunits on immune cells known to be important for clearance of viral infections, splenocytes from unimmunized or VV Western Reserve (VV-WR) strain infected BALB/c WT mice were analyzed using flow cytometry during the peak (i.e. 7 days) of anti-viral immunity. Cell surface IL-13Rα1, γc and intracellular IL-13Rα2 expression levels on gated B220^+^, CD4^+^, CD8^+^, DX5^+^ (natural killer cells) and CD11c_high_ I-A^d^_high_ (dendritic cells (DCs)) splenocytes from VV-WR infected mice and unimmunized mice were similar (figure 1). Cell surface IL-13Rα2 expression was not detectable above background expression levels in the cell subsets shown in figure 1 (data not shown). Interestingly, cell surface IL-4Rα expression was up-regulated on CD11c_high_ I-A^d^_high_ cells and down-regulated on CD4^+^ T cells and CD8^+^ T cells as a consequence of VV infection (figure 1). Down-regulation of IL-4Rα was not uniformly observed on all CD4^+^ or CD8^+^ T cells following VV infection with some cells retaining similar IL-4Rα expression levels to that observed in unimmunized mice.

### Down-regulation of IL-4Rα expression correlates with the magnitude of effector CD8^+^ T cells

**Figure 2:**
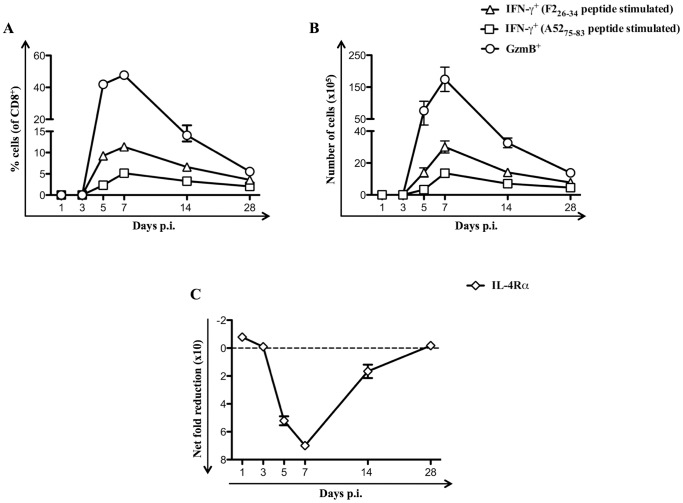
Reduction in IL-4Rα expression correlates with magnitude of anti-viral effector responses on CD8^+^ T cells. Unimmunized or VV-WR infected BALB/c WT mice (n = 4 per group) were sacrificed at the indicated time points and splenocytes used for flow cytometry analysis or for ICS following A52_75–83_ or F2_26–34_ *in vitro* peptide stimulation of splenocytes as described in the materials and methods. A and B, The mean (n = 4) percentage of CD8^+^ splenocytes (A) and the total number of CD8^+^ splenocytes (B) from VV-WR infected mice that expressed GzmB or IFN-γ following *in vitro* peptide stimulation. C, The kinetics of the mean (n = 4) net fold reduction in cell surface expression of IL-4Rα on gated CD8^+^ splenocytes from VV-WR infected mice relative to unimmunized mice. Net fold reduction was calculated using MFI values as described in the materials and methods. The data shown are representative of at least two independent experiments and the error bars depict the SEM.

To clearly understand the mechanisms responsible for mediating down-regulation of cell surface IL-4Rα expression on CD8^+^ T cells following VV infection, the kinetics of down-regulation of cell surface IL-4Rα expression on CD8^+^ T cells from the spleens of BALB/c WT mice infected with VV-WR was monitored. Using intracellular cytokine staining (ICS) and granzyme B (GzmB) expression assays, data revealed that VV-specific effector CD8^+^ T cells emerged on day 5 post-infection (p.i.), peaked on day 7 p.i. and gradually declined from days 14–28 p.i. with VV-WR (figure 2A and 2B). Similarly, reduction in cell surface IL-4Rα expression on CD8^+^ T cells following VV-WR infection was detected on day 5 p.i., peaked on day 7 p.i. and declined from days 14–28 p.i. (figure 2C). Thus, the magnitude of down-regulation of IL-4Rα expression on CD8^+^ T cells correlated with the increase in the magnitude of the anti-viral effector CD8^+^ T responses following VV infection.

### Down-regulation of IL-4Rα is restricted to virus-specific CD8^+^ T cells

**Figure 3:**
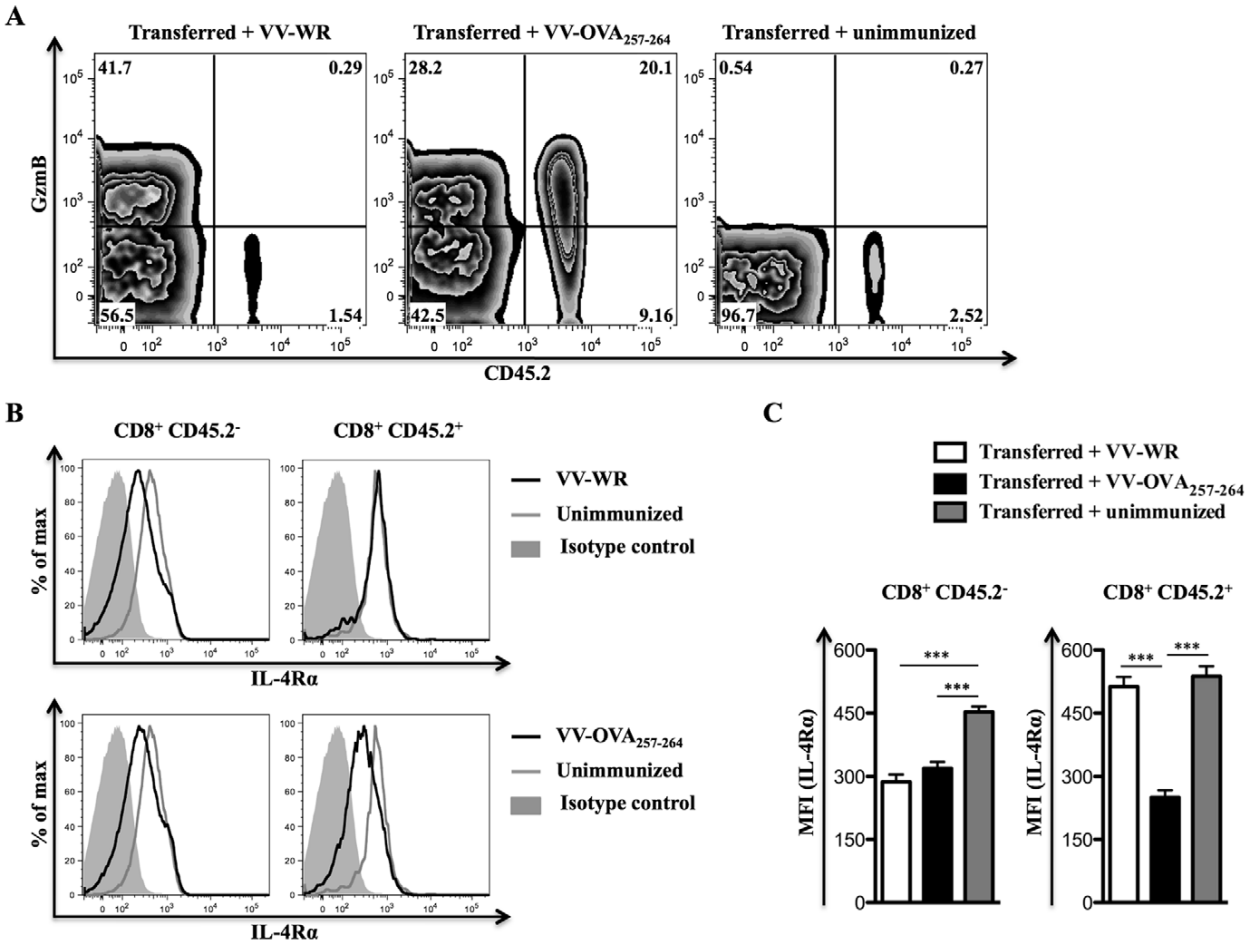
Cell surface down-regulation of IL-4Rα specifically occurs on activated CD8^+^ T cells. Naïve C57BL/6.SJL (CD45.1^+^; CD45.2^−^) mice (n = 6 group) that received 10×10^6^ C57BL/6 OT-I splenocytes (CD45.2^+^) i.v. were kept unimmunized or infected i.p. with 5×10^6^ PFU of VV-WR or VV-OVA_257–264_ for 7 days prior to sacrifice and flow cytometry analysis. A, Representative contour plots showing cell surface CD45.2 and intracellular GzmB expression on gated CD8^+^ splenocytes from a recipient mouse of the indicated group. B, Representative histogram plots showing cell surface IL-4Rα expression on gated CD8^+^ CD45.2^−^ (left column of plots) or CD8^+^ CD45.2^+^ (right column of plots) splenocytes from a recipient mouse kept unimmunized, infected with VV-WR or VV-OVA_257–264_. C, Mean (n = 6) MFI representing cell surface IL-4Rα expression on gated CD8^+^ CD45.2^−^ or CD8^+^ CD45.2^+^ splenocytes from recipient mice of the indicated group. One-way ANOVA (Tukey's Multiple Comparison) was used for testing significance of the data (*** - p<0.001). Similar results have been obtained in three independent experiments and the error bars depict the SEM.

Next the OT-I T cell receptor transgenic system was used to show whether IL-4Rα down-regulation was restricted to CD8^+^ T cells responding to virus. Splenocytes from OT-I mice (CD45.2^+^) were transferred intravenously (i.v.) into congenic C57BL/6.SJL (CD45.1^+^; CD45.2^−^) recipient mice prior to infection of these mice with VV-WR expressing the ovalbumin peptide SIINFEKL (VV-OVA_257–264_) or VV-WR, which does not express OVA_257–264_ (SIINFEKL) epitope. CD8^+^ T cells from OT-I mice almost exclusively recognize K^b^OVA_257–264_ and so should only be primed by VV-OVA_257–264_ and not VV-WR. This was confirmed using GzmB as a marker of activation (figure 3A). IL-4Rα levels were also measured (figure 3B and 3C), and down-regulation of this receptor on OT-I cells (CD45.2^+^) was only seen in mice infected with VV-OVA_257–264_. By contrast IL-4Rα levels were reduced on recipient (CD45.2^−^) CD8^+^ T cells irrespective of the strain of virus. Thus, IL-4Rα levels were only reduced on activated virus-specific CD8^+^ T cells.

### Down-regulation of IL-4Rα is a general property of activated anti-viral CD8^+^ T cells

**Figure 4:**
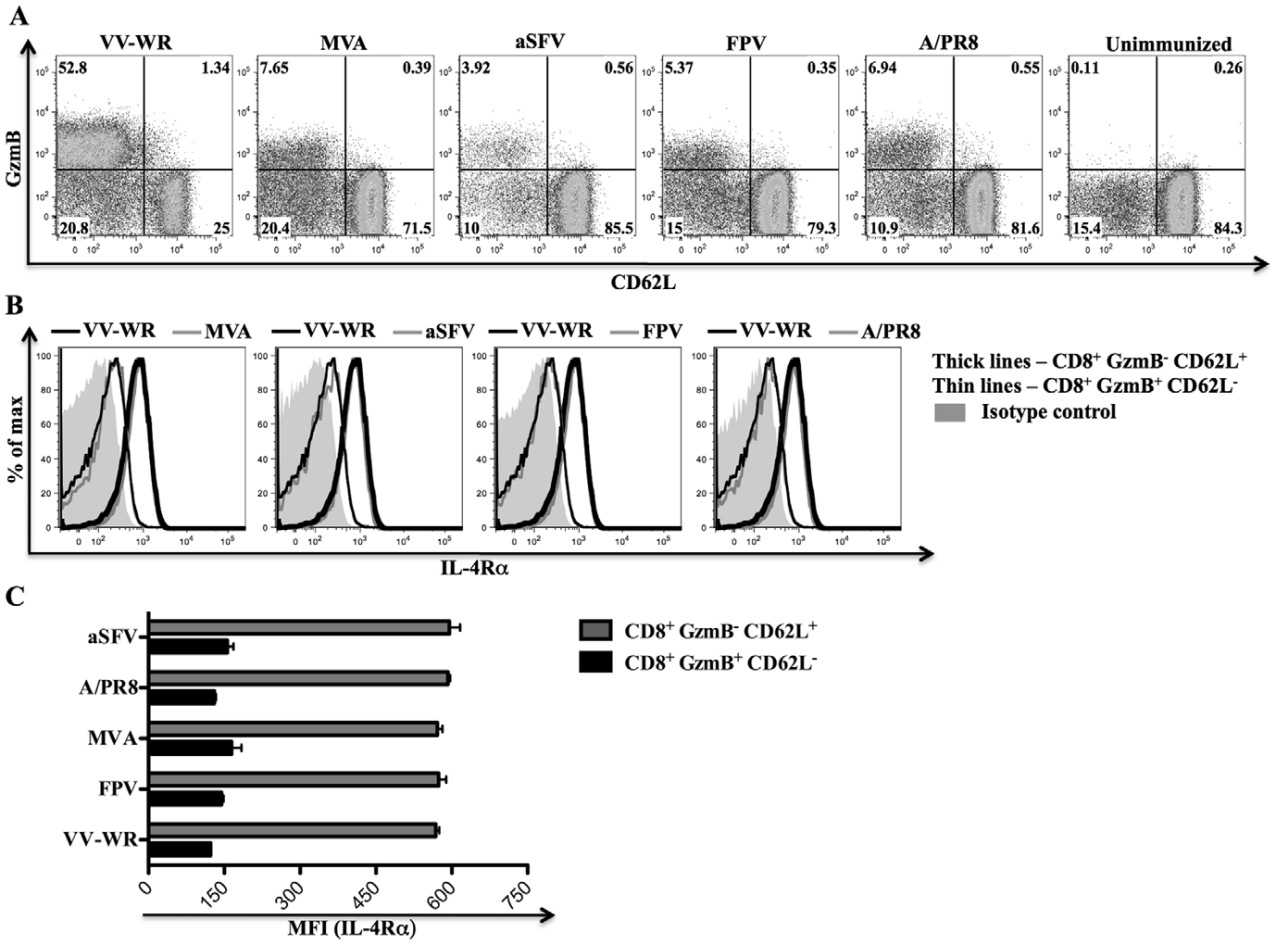
Down-regulation of IL-4Rα is a general feature of activated CD8^+^ T cells following virus infection. BALB/c WT mice (n = 5) were infected i.p. with 3×10^6^ PFU of the indicated viruses or kept unimmunized for 7 days prior to sacrifice and flow cytometry analysis. A, Dot plots showing cell surface CD62L expression and intracellular GzmB expression on gated CD8^+^ splenocytes from a representative mouse of the indicated group. B, Representative histogram plots showing cell surface IL-4Rα expression on the indicated CD8^+^ splenocyte subset from a representative mouse infected with the indicated virus. C, Mean (n = 5) MFI representing cell surface IL-4Rα expression on the indicated splenocyte subset from mice infected with the indicated viruses. Similar results have been obtained in three independent experiments and the error bars shown depict the SEM.

To evaluate whether down-regulation of cell surface IL-4Rα expression on activated CD8^+^ T cells is specific to VV infections, we analyzed IL-4Rα expression on effector (GzmB^+^ CD62L^−^) and naïve (GzmB^−^ CD62L^+^) CD8^+^ T cells as described in Yuen *et al* [23] following infection of BALB/c WT mice with VV-WR, modified vaccinia Ankara (MVA), avirulent Semliki Forest virus (aSFV), fowlpox virus (FPV) or A/PR8 (H1N1) influenza virus (figure 4A). Cell surface IL-4Rα expression on effector (GzmB^+^ CD62L^−^) CD8^+^ T cells compared to naïve bystander (GzmB^−^ CD62L^+^) CD8^+^ T cells was significantly lower in all the mice infected with the different viruses (figure 4B and 4C). There were no significant differences between the levels of cell surface IL-4Rα expression on effector CD8^+^ T cells or naïve CD8^+^ T cells that developed following VV-WR infection and other viral infections (figure 4B and 4C). Therefore, down-regulation of cell surface IL-4Rα expression on activated CD8^+^ T cells is a general feature of virus infections *in vivo*.

### Importance of IL-4, IL-13, IFN-γ and STAT6 in regulating IL-4Rα expression on CD8^+^ T cells following virus infection

**Figure 5:**
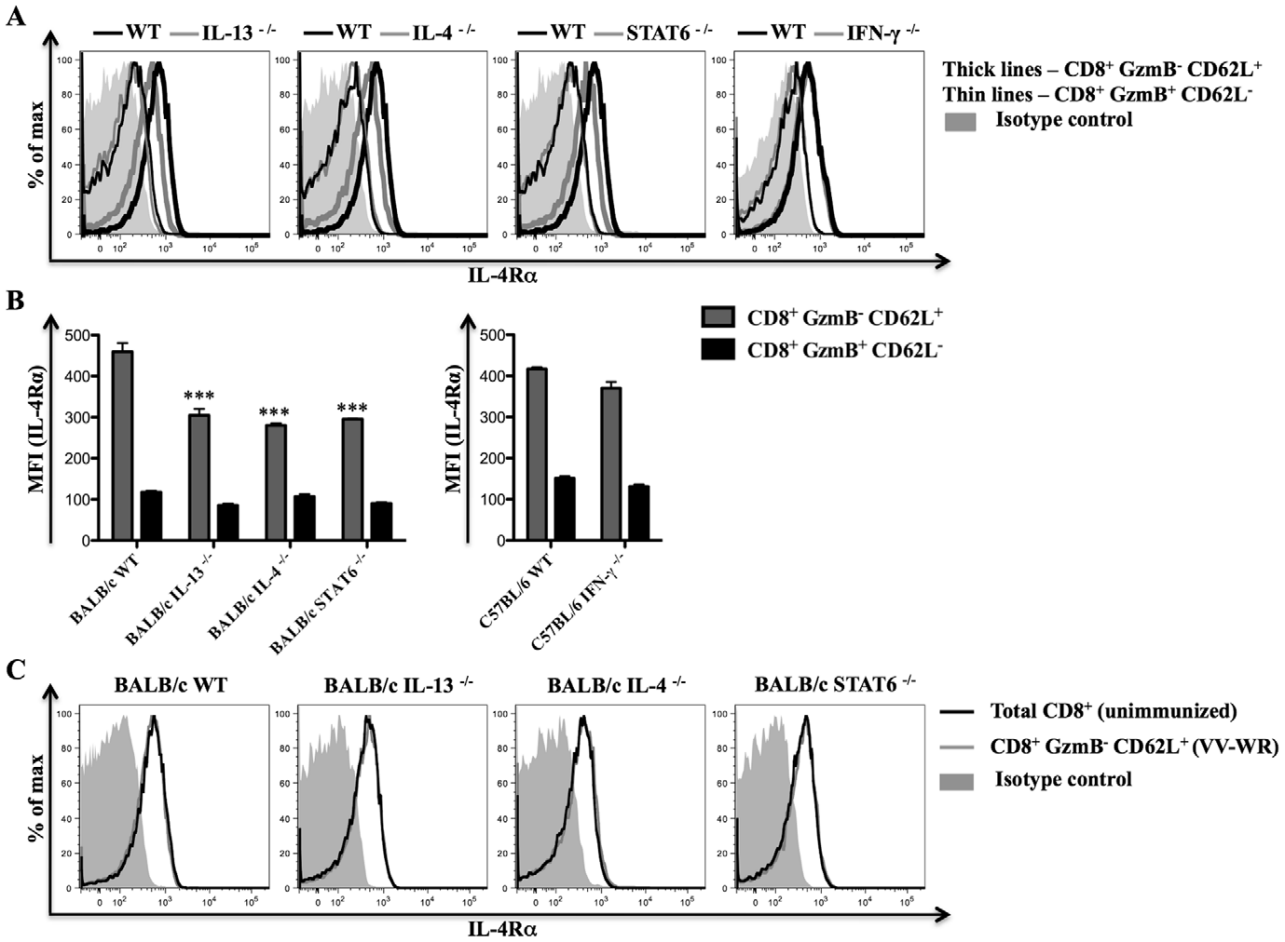
IL-4, IL-13 and STAT6 regulate IL-4Rα expression on CD8^+^ T cells following VV-WR infection. Gene knockout mice (n = 5) and respective littermate WT control mice (n = 5) were infected with VV-WR for 7 days or kept unimmunized prior to sacrifice and analysis using flow cytometry. A, Representative histogram plots showing cell surface IL-4Rα expression on the indicated CD8^+^ splenocyte subset from a gene knockout mouse or a littermate WT control mouse. B, Mean (n = 5) MFI representing cell surface IL-4Rα expression on the indicated CD8^+^ splenocyte subset from mice of the indicated genetic background infected with VV-WR. C, Representative histogram plots showing cell surface IL-4Rα expression on the indicated CD8^+^ splenocyte subset from an unimmunized mouse or VV-WR infected mouse belonging to the indicated genetic background. All the results shown are representative of at least two independent experiments. Error bars when shown depict the SEM and one-way ANOVA (Tukey's Multiple Comparison) was used to determine the statistical significance of the data relative to WT control mice (*** - p<0.001).

IL-4, IL-13 and IFN-γ are cytokines that play a role in regulating IL-4Rα expression on cells [12], [24]. Thus, we next determined whether down-regulation of cell surface IL-4Rα expression on anti-viral effector CD8^+^ T cells was dependent on IL-4, IL-13, STAT6 or IFN-γ. For this purpose, IL-4Rα expression was analyzed on effector (GzmB^+^ CD62L^−^) and naïve (GzmB^−^ CD62L^+^) CD8^+^ T cells from relevant gene knockout mice and littermate WT control mice infected with VV-WR. Cell surface IL-4Rα expression on naïve CD8^+^ T cells, but not effector CD8^+^ T cells was significantly lower in BALB/c IL-4 ^−/−^, BALB/c IL-13 ^−/−^ and BALB/c STAT6 ^−/−^ mice compared to BALB/c WT control mice (figure 5A and 5B). We also examined whether a similar trend applied to CD4^+^ T cells in these experiments. However, when comparing cell surface IL-4Rα expression on naïve (CD62L^hi^ CD44^lo^) or effector (CD62L^lo^ CD44^hi^) CD4^+^ T cells from WT mice and gene knockout mice, no differences were observed (data not shown).

In these experiments, we also compared IL-4Rα expression levels on naïve CD8^+^ T cells from VV-WR infected mice and unimmunized mice. This was done to investigate the possibility that VV-WR infection induced IL-4 and/or IL-13 to up-regulate IL-4Rα expression on naïve bystander CD8^+^ T cells similar to what has been reported on naïve bystander CD4^+^ T cells responding to IL-4 following parasitic infection [22]. However, naïve CD8^+^ T cells from VV-WR infected BALB/c IL-13 ^−/−^, BALB/c IL-4 ^−/−^, BALB/c STAT6 ^−/−^, or BALB/c WT mice expressed similar levels of cell surface IL-4Rα to that of naïve CD8^+^ T cells from unimmunized mice belonging to the respective genetic background (figure 5C). This suggests that VV-WR infection was not inducing significant levels of IL-4 and/or IL-13 to up-regulate IL-4Rα expression on naïve CD8^+^ T cells. In contrast to the knockout mice described above, no difference in IL-4Rα expression levels were seen on any CD8^+^ T cells from C57BL/6 IFN-γ ^−/−^ compared to C57BL/6 WT controls (figure 5A and 5B). Collectively, data suggest that IL-4, IL-13 and STAT6 are required for maintaining high levels of IL-4Rα expression on naïve CD8^+^ T cells.

### Increased IL-4 during virus infection induces higher levels of IL-4Rα on CD8^+^ T cells

**Figure 6:**
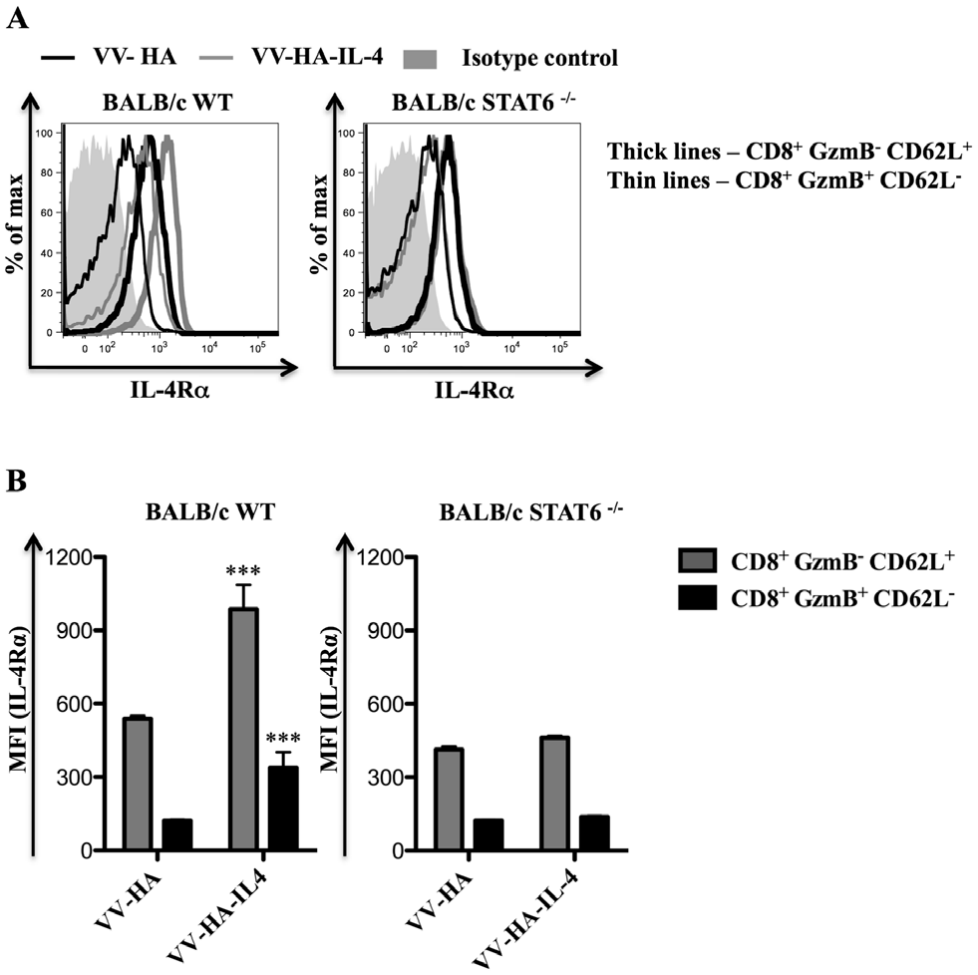
IL-4Rα is up-regulated on CD8^+^ T cells in a STAT6 dependent manner following VV-HA-IL-4 infection. BALB/c WT mice (n = 5) or BALB/c STAT6 ^−/−^ mice (n = 5) were infected with 5×10^6^ PFU of VV-HA control, VV-HA-IL-4 or kept unimmunized for 7 days prior to sacrifice and analysis using flow cytometry. A and B, Representative histogram plots showing cell surface IL-4Rα expression (A) and the mean (n = 5) MFI of cell surface IL-4Rα expression (B) on the indicated CD8^+^ splenocyte subset from infected mice of the indicated genetic background. The data shown is representative of two independent experiments and the error bars depict the SEM. One-way ANOVA (Tukey's Multiple Comparison) was used to determine the statistical significance of the data relative to respective cell subset from VV-HA infected mice (*** - p<0.001).

Next we investigated whether IL-4Rα levels on CD8^+^ T cells were fixed/unchanged, or responsive to IL-4 during infection. For these studies VV co-expressing murine IL-4 and hemagglutinin (HA), namely VV-HA-IL-4 [25], and the control VV co-expressing HA (VV-HA) were used. Strikingly, IL-4Rα expression was higher in BALB/c WT mice infected with VV-HA-IL-4 relative to VV-HA infection when comparing naïve (GzmB^−^ CD62L^+^) or effector (GzmB^+^ CD62L^−^) CD8^+^ T cells (figure 6). However, when the same experiment was performed using BALB/c STAT6 ^−/−^ mice, no difference in IL-4Rα expression was observed (figure 6). Data indicate that the amount of IL-4 available during virus infection can regulate the expression of IL-4Rα on CD8^+^ T cells in a STAT6 dependent manner.

### IL-4 and IL-13 regulate the establishment of IFN-γ^+^ TNF-α^+^ producing effector CD8^+^ T cells following virus infection

**Figure 7:**
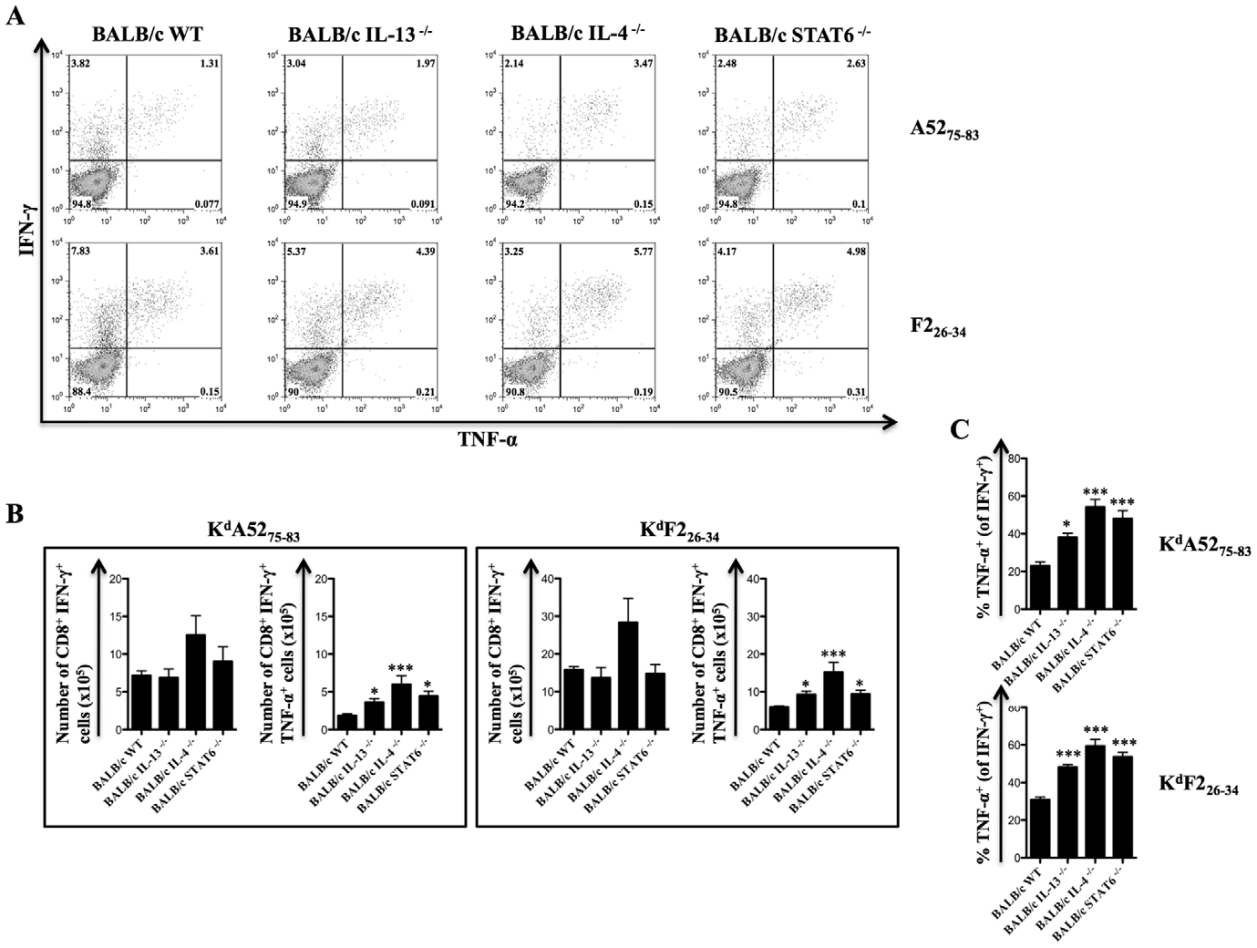
IL-4 and IL-13 dampen polyfunctional (IFN-γ^+^ TNF-α^+^) CD8^+^ T cell numbers following VV-WR infection. BALB/c IL-13 ^−/−^, BALB/c IL-4 ^−/−^, BALB/c STAT6 ^−/−^ and BALB/c WT control mice (n = 6 per group) were infected with 3×10^6^ PFU of VV-WR or kept as unimmunized controls for 7 days prior to sacrifice and analysis using ICS after *in vitro* peptide stimulation. A, Representative dot plots showing IFN-γ and TNF-α expression on gated CD8^+^ splenocytes from VV-WR infected mice of the indicated genetic background following *in vitro* stimulation of splenocytes with the indicated peptides. B, Mean (n = 6) total number of K^d^A52_75–83_ or K^d^F2_26–34_ specific CD8^+^ IFN-γ^+^ or CD8^+^ IFN-γ^+^ TNF-α^+^ splenocytes from the indicated mice infected with VV-WR. C, Mean (n = 6) proportion of K^d^A52_75–83_ or K^d^F2_26–34_ specific CD8^+^ IFN-γ^+^ splenocytes that also produced TNF-α from the indicated mice infected with VV-WR. The data presented in all panels are representative of at least two independent. Error bars depict the SEM and statistical significance was determined using a one-way ANOVA (Tukey's Multiple Comparison) relative to WT control mice (* - p<0.05; *** - p<0.001).

High avidity virus-specific CD8^+^ T cells express elevated levels of both IFN-γ and TNF-α [8], [26]. To evaluate whether differential regulation of cell surface IL-4Rα expression on CD8^+^ T cells may play a role in regulating CD8^+^ T cell functional quality, IFN-γ and TNF-α production were measured using ICS following i*n vitro* A52_75–83_ or F2_26–34_ peptide stimulation of splenocytes from VV infected BALB/c WT mice and gene knockout mice. Amongst the gene knockout mice examined only BALB/c IL-4 ^−/−^ mice developed greater numbers of IFN-γ expressing K^d^A52_75–83_ or K^d^F2_26–34_ specific-CD8^+^ T cells compared to BALB/c WT control mice following VV-WR infection (figure 7B). However, the enhancement in cell numbers in this instance was not robust enough to reach statistical significance (figure 7B). The number of IFN-γ^+^ TNF-α^+^ K^d^A52_75–83_ or K^d^F2L_26–34_ specific-CD8^+^ T cells and the proportion of K^d^A52_75–83_ or K^d^F2L_26–34_ specific-CD8^+^ T cells that expressed TNF-α in addition to IFN-γ was higher in VV-WR infected BALB/c IL-13 ^−/−^, BALB/c IL-4 ^−/−^ and BALB/c IL-STAT6 ^−/−^ mice compared to BALB/c WT control mice (figure 7). Given that IL-4Rα expression on naïve CD8^+^ T cells was lower in BALB/c IL-13 ^−/−^, BALB/c IL-4 ^−/−^ and BALB/c IL-STAT6 ^−/−^ mice compared to BALB/c WT mice (figure 5), in these studies, lower IL-4Rα expression on naïve CD8^+^ T cells correlated with the enhancement in the anti-viral IFN-γ^+^ TNF-α^+^ CD8^+^ T cell responses.

**Figure 8:**
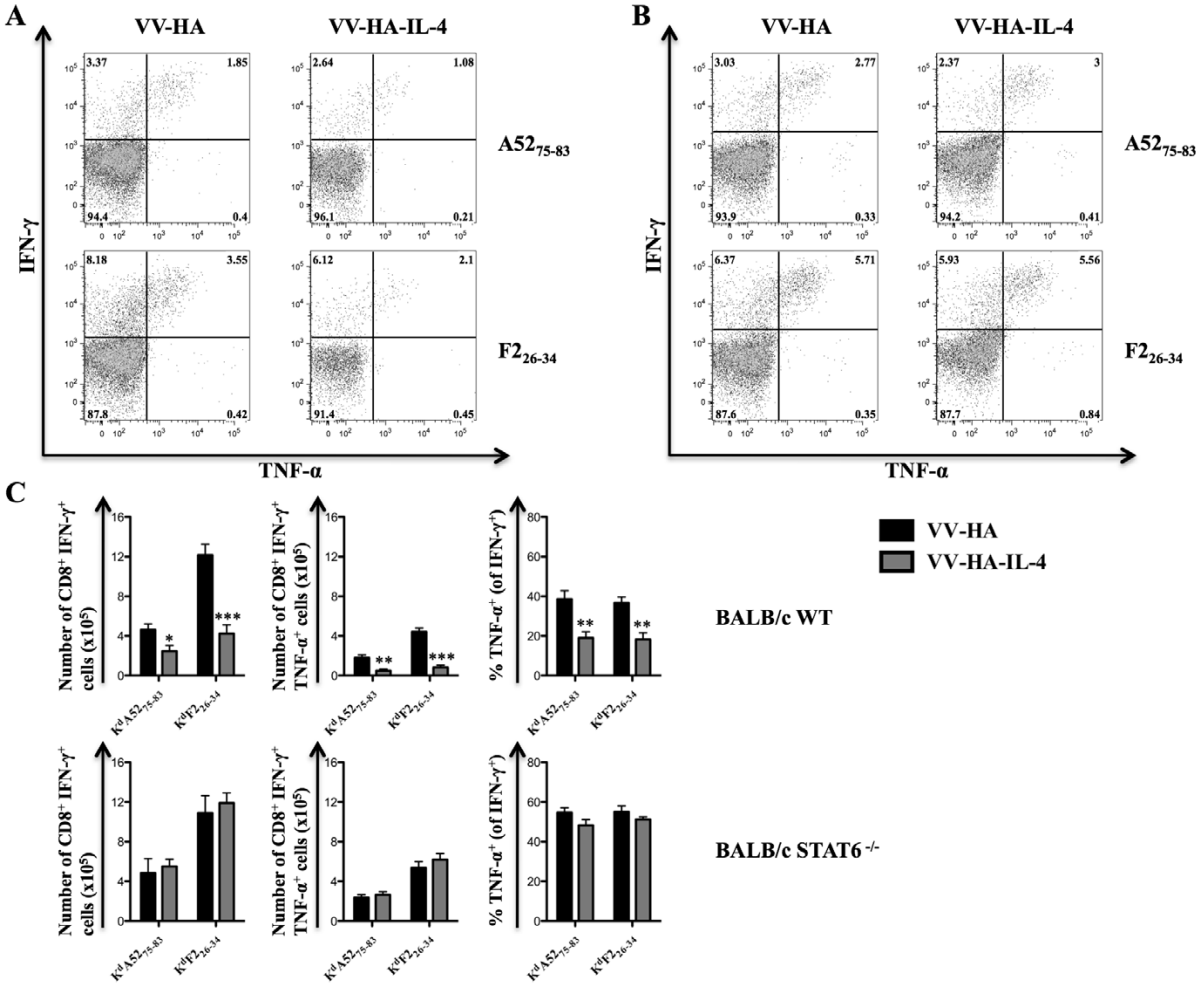
STAT6 is required for IL-4 mediated attrition of VV-specific CD8^+^ T cell responses. BALB/c STAT6 ^−/−^ (n = 4) and BALB/c WT control mice (n = 5) were infected with 5×10^6^ PFU of VV-HA, VV-HA-IL-4 or kept unimmunized for 7 days prior to sacrifice and analysis using ICS after *in vitro* peptide stimulation. A and B, Representative dot plots showing IFN-γ and TNF-α expression on gated CD8^+^ splenocytes from infected BALB/c WT (A) or BALB/c STAT6 ^−/−^ (B) mice following *in vitro* stimulation of splenocytes with the indicated peptides. C, The mean (n = 4–5) number of cytokine producing K^d^A52_75–83_ or K^d^F2_26–34_ specific CD8^+^ splenocytes and the mean (n = 4–5) proportion of CD8^+^ splenocytes that produced TNF-α in addition to IFN-γ from BALB/c WT (top row of plots) or BALB/c STAT6 ^−/−^ (bottom row of plots) mice infected with the indicated virus. The data are representative of two independent experiments and the error bars depict the SEM. One-way ANOVA (Tukey's Multiple Comparison) was used to determine statistical significance of the data relative to VV-HA infected mice (* - p<0.05; ** - p<0.01; *** - p<0.001).

Consistent with the above findings, infection of BALB/c WT mice with VV-HA-IL-4 compared to VV-HA control infection significantly reduced the numbers of IFN-γ^+^ and IFN-γ^+^ TNF-α^+^ K^d^A52_75–83_ or K^d^F2_26–34_ specific-CD8^+^ T cells (figure 8A and 8C). The proportion of K^d^A52_75–83_ or K^d^F2_26–34_ specific-CD8^+^ T cells that expressed TNF-α in addition to IFN-γ was also significantly reduced in BALB/c WT mice infected with VV-HA-IL-4 compared to VV-HA (figure 8A and 8C). Unlike that observed with BALB/c WT mice, infection of BALB/c STAT6 ^−/−^ mice with VV-HA-IL-4 compared to VV-HA control did not impair the IFN-γ and TNF-α cytokine production capacity of K^d^A52_75–83_ or K^d^F2_26–34_ specific-CD8^+^ T cells (figure 8B and 8C). Thus, elevation of IL-4Rα expression on CD8^+^ T cells during virus infection strongly correlated with the reduction of anti-viral IFN-γ^+^ TNF-α^+^ CD8^+^ T cell responses.

## Discussion

Our results clearly indicated that amongst the different IL-4/IL-13 receptor components only cell surface IL-4Rα expression was significantly down-regulated on activated CD8^+^ T cells following virus infection. IL-4, IL-13 and STAT6 were required to elevate IL-4Rα expression on naïve CD8^+^ T cells, but not IFN-γ. VV-HA-IL-4 infection studies showed that IL-4 and STAT6 was required to up-regulate IL-4Rα expression on naïve and effector CD8^+^ T cells. In all these studies higher IL-4Rα expression on CD8^+^ T cells strongly correlated with the reduction in polyfunctional or IFN-γ^+^ TNF-α^+^ anti-viral CD8^+^ T cells. Thus, we propose that regulation of IL-4Rα expression during virus infection plays an important role in regulating the quality of anti-viral CD8^+^ T cell immunity.

Contrary to other studies [12], our data showed that IL-13Rα1 is ubiquitously expressed on CD4^+^ T cells, CD8^+^ T cells, B cells, natural killer cells and DCs. Similar ubiquitous expression on immune cells was also observed with respect to IL-4Rα, γc, and IL-13Rα2. However, following virus infection only IL-4Rα was notably differentially regulated on certain immune cell subsets (i.e. CD4^+^ T cells, CD8^+^ T cells and DCs). The mechanisms responsible for regulating IL-4Rα expression on CD4^+^ T cells and DCs following virus infection were not investigated in this study and warrants further investigation especially given the importance of these cell subsets in driving immune responses that help control intracellular pathogens [4], [27].

In an acute lymphocytic choriomeningitis virus (LCMV) adoptive transfer model, Wherry *et al* [28] have reported changes in many cellular markers, including IL-4Rα expression on transferred LCMV DbGP33-specific transgenic P14 CD8^+^ T cells. In their model, IL-4Rα expression was down-regulated on LCMV DbGP33-specific CD8^+^ T cells following acute and chronic LCMV infection. These findings are consistent with the findings of the current study. However, unlike the current study, Wherry *et al* [28] did not investigate the factors responsible for regulating IL-4Rα expression on polyclonal CD8^+^ T cells or the implications of regulating this receptor on CD8^+^ T cell functionality following virus infection in WT mice.

IL-4 can induce the activation of STAT1, STAT3, STAT5 and STAT6 on naïve and activated CD8^+^ T cells *in vitro* [29], but our infections studies with VV-HA-IL-4 indicated that STAT6 was indispensible for IL-4 mediated up-regulation of cell surface IL-4Rα expression on naïve and effector CD8^+^ T cells. Even in naïve gene knockout mice STAT6 signaling, which appeared to be optimal in the presence of both IL-4 and IL-13, was required to maintain high levels of cell surface IL-4Rα expression on CD8^+^ T cells. Thus, our data indicated that IL-4 and/or IL-13 signaling through STAT6 was important in maintaining high levels of IL-4Rα expression on naïve CD8^+^ T cells even when no pathogen is encountered. Furthermore, this signaling mechanism also appears to be important in elevating IL-4Rα expression on naïve and effector CD8^+^ T cells *in vivo* following virus infection. However, a caveat in this interpretation is that deficiency of STAT6 in other cells may play a role in regulating IL-4Rα expression levels on CD8^+^ T cells.

Previous infection studies with VV-HA-IL-4 or recombinant ectromelia virus encoding IL-4 suggested that IL-4 dampened the effector function capacity of anti-viral CD8^+^ T cells and enhanced viral pathogenesis [25], [30]. In chronic HIV progressors, HIV-specific CD8^+^ T cells with reduced cytolytic capacity have been reported to express IL-4 [31]. In our laboratory, following HIV-1 pox-viral prime-boost vaccination, both IL-4 and IL-13 were found to dampen the avidity of HIV-specific CD8^+^ T cells [7]. However, none of the above studies have monitored the expression of IL-4Rα in conjunction with the anti-viral effector functions on CD8^+^ T cells. We have addressed this in the current study and our data suggest that enhancing CD8^+^ T cell responsiveness to IL-4 and/or IL-13 via STAT6 dependent up-regulation of IL-4Rα expression can exacerbate the quality (IFN-γ and TNF-α cytokine production) of anti-viral CD8^+^ T cell immunity.

Paradoxical to these studies, we have also shown that HIV-specific CD8^+^ T cells that developed in IL-4Rα ^−/−^ mice following HIV-1 prime-boost vaccination expressed high levels of IL-4 and IL-13, but low levels of IFN-γ [7]. Other studies using IL-4Rα ^−/−^ mice have also shown that IL-4Rα is required for the maintenance of optimal CD8^+^ T cell cytotoxicity, IFN-γ production and memory responses [32]–[33]. It is highly likely that in IL-4Rα ^−/−^ mice, other compensatory mechanisms may play a role in dampening CD8^+^ T cell functionality as Mohrs *et al* [34] have shown that T helper 2 differentiation can still occur in IL-4Rα ^−/−^ mice *in vivo*. Identification of these compensatory mechanisms may not only help understand mechanisms important for regulating T cell quality, but also help reconcile the paradoxical findings discussed above.

The exact mechanisms as to how IL-4 and/or IL-13 mediated elevation of IL-4Rα expression on CD8^+^ T cells act to dampen IFN-γ and TNF-α cytokine production by anti-viral CD8^+^ T cells was not addressed in this study. It is possible that activation of suppressor of cytokine signaling (SOCS)-1 and -3 on anti-viral CD8^+^ T cells is important for this effect as IL-4 and IL-13 mediated activation of these transcription factors have been shown to dampen IFN-γ and TNF-α cytokine production by keratinocytes [35]. IL-4 has also been shown to down-regulate the CD8 co-receptor expression levels on antigen-specific CD8^+^ T cells, which dampens the functionality of these cells [9]–[11]. Therefore, IL-4 and/or IL-13 mediated regulation of the CD8 co-receptor expression levels on anti-viral CD8^+^ T cells may also play a role in regulating anti-viral cytokine production and this is currently being evaluated in an HIV-1 pox viral prime-boost vaccination model (Wijesundara *et al* manuscript in preparation).

To our understanding, this is the first study to evaluate how the different cellular receptor components for IL-4 and IL-13 are regulated on CD8^+^ T cells following virus infection. Our data suggested that differential regulation of IL-4Rα, unlike other IL-4/IL-13 receptor components (i.e. γc, IL-13Rα1 and IL-13Rα2) play a more critical role in determining the responsiveness of CD8^+^ T cells to IL-4 and/or IL-13 in order to regulate the quality of anti-viral CD8^+^ T cell immunity. This is consistent with our previous studies where IL-4 and IL-13 were shown to play an important role in modulating the avidity of HIV-specific CD8^+^ T cell responses following prime-boost vaccination [7], [8]. Thus, we believe that the current findings could be exploited to design more effective pox viral-vectored vaccines against chronic infections such as HIV-1 where robust CD8^+^ T cell immunity is required for protection. Furthermore, given that IL-4Rα is constitutively expressed on T cells, it may easily be used as a novel biomarker to assess T cell quality following vaccination.

## Materials and Methods

### Mice

Pathogen-free 6–8 weeks old female BALB/c WT, BALB/c IL-4 ^−/−^, BALB/c IL-13 ^−/−^, BALB/c STAT6 ^−/−^, C57BL/6, C57BL/6.SJL (CD45.1) and T cell receptor transgenic C57BL/6 OT-I (CD45.2) mice were all purchased from the Australian Phenomics Facility, the Australian National University. Associate Professor Guna Karupiah kindly provided the C57BL/6 IFN-γ ^−/−^ mice used in this study.

### Ethics statement

All animals were maintained and experiments were performed in accordance with the Australian NHMRC guidelines within the Australian Code of Practice for the Care and Use of Animals for Scientific Purposes and in accordance with guidelines approved by the Australian National University Animal Experimentation and Ethics Committee (AEEC), protocol numbers JIG 74.09 and A2011/018. All animals were monitored daily, infected mice were scored for signs of illness and weight loss. Animals were ethically sacrificed using cervical dislocation in accordance with the above AEEC approved protocols.

### Peptide synthesis

VV immunodominant A52_75–83_ (KYGRLFNEI) and F2L_26–34_ (SPYAAGYDL) peptides were synthesized by the Biomolecular Resource Facility, the John Curtin School of Medical Research.

### Virus and infection

VV-WR strain and recombinant VV-OVA_257–264_ were grown and titrated in 143B cells. Recombinant VV-HA-IL-4 and the control VV-HA were prepared as described in Sharma *et al* [25]. These stocks were kindly provided by Professor Alistair Ramsay. FPV were grown and titrated in chick embryonic epithelial cells. Dr. David Boyle kindly provided the FPV parent stocks. MVA, aSFV and A/PR8 (H1N1) influenza virus stocks used in this study were gifts from Associate Professor Guna Karupiah, Dr. Mohammed Alsharifi and Dr. Yoichi Furuya, respectively.

All virus infections in age- and sex-matched mice were conducted intraperitoneally (i.p.) at a dose of 3×10^6^ plaque forming units (PFU)/mouse or 5×10^6^ PFU/mouse for a period of 7 days unless otherwise stated.

### Adoptive cell transfer

Red blood cells (RBC)-depleted splenocytes from 8 weeks old C57BL/6 OT-I mice were injected i.v. (10×10^6^ cells in 200 µl of phosphate buffer saline) into a lateral tail vein of 8 weeks old recipient C57BL/6.SJL mice. Subsequently, the recipient mice were rested overnight and infected i.p. with 5×10^6^ PFU/mouse of VV-WR control or VV-OVA_257–264_.

### Flow cytometry

The following monoclonal antibodies against mouse antigens and the respective isotype controls for CD8α, CD4, CD45R (B220), CD124 (IL-4Rα), CD132 (γc), CD49b (DX5), CD62L, CD11c and TNF-α were all obtained from Becton Dickinson (BD) Biosciences, USA. Monoclonal antibodies against mouse GzmB, CD45.2 and I-A^d^ were obtained from BioLegend, USA and monoclonal antibodies against mouse CD213a (IL-13Rα1) and IFN-γ were obtained from eBioscience, USA. These antibodies were used as purified, fluorescein isothiocyanate, phycoerythrin, peridinin chlorophyll protein, pacific blue, or allophycocyanin conjugates. Secondary fluorescein isothiocyanate conjugated anti-goat IgG (Jackson Immuno Research, USA) was used to detect purified polyclonal goat IgG anti-mouse IL-13Rα2 (R&D systems, USA) binding to splenocytes.

In all flow cytometry-based studies RBC-depleted splenocytes were used. Cell surface staining and intracellular staining were performed using the respective method described in Ranasinghe *et al* [36]. Briefly, for cell surface staining 1–2×10^6^ cells were incubated 30 minutes at 4°C in the presence of purified or fluorochrome conjugated antibodies diluted in phosphate buffer saline containing 1% fetal calf serum (FCS). Subsequently, samples were washed twice using phosphate buffer saline containing 1% FCS, fixed in 0.5% paraformaldehyde, prior to analysis.

In all the experiments described here, intracellular GzmB staining of cell surface stained samples were conducted using freshly isolated RBC-depleted splenocytes that were not stimulated with peptides. For IFN-γ and TNF-α ICS, 1×10^6^ cells in 200 µl of Roswell Park Memorial Institute culture medium (Invitrogen, Australia) containing 10% FCS were seeded in 96 well U-bottom plates in the presence or absence of 0.1 µg/ml of A52_75–83_ or F2_26–34_ peptides. Subsequently, peptide stimulated or unstimulated cultures were left for 1 hour at 37°C + 5% CO_2_ prior to addition of 1× Brefeldin A (eBioscience, USA). The cultures were then left for further 4 hours at 37°C+5% CO_2_ prior to conducting cell surface staining as described before.

Cell surface stained samples were fixed and permeabilized using commercial intracellular fixation and permeabilization buffers according to the manufacturer's protocol (eBioscience, USA). Subsequently, permeabilized cells were incubated 30 minutes at 4°C in the presence of purified or fluorochrome conjugated antibodies diluted in 1× permeabilization buffer (eBioscience, USA). Intracellular stained samples were washed twice using phosphate buffer saline containing 1% FCS, fixed in 0.5% paraformaldehyde prior to analysis. All cell surface and intracellular stained samples were analyzed using FACS Calibur (BD Biosciences, USA) or BD LSR II (BD Biosciences, USA) machine. Flow cytometry plots of the analyzed data were constructed using the FlowJo Tree Star software (version 8.7.1).

### Statistical analysis

Net fold reduction of cell surface IL-4Rα expression was calculated using mean fluorescent intensity (MFI) obtained from flow cytometry on gated CD8^+^ splenocytes from unimmunized or VV-WR infected mice as follows: ((MFI (IL-4Rα)_unimmunized_−MFI (IL-4Rα)_VV-WR_)/MFI (IL-4Rα)_unimmunized_). All the data presented in this study have been reproduced in at least two independent experiments. The data plotted in all the graphs shown represent the mean and the error bars depict the standard error of the mean (SEM). Statistical significance of the data and the p values were calculated using the Graph InStat software (version 3.10). In all statistical significance analysis, a student's unpaired t-test or one-way ANOVA (Tukey's Multiple Comparison post-hoc test) was used. The p values are denoted as follows: * - p<0.05, ** - p<0.01, and *** - p<0.001.
